# Technical Developments and Clinical Use of Telemedicine in Sleep Medicine

**DOI:** 10.3390/jcm5120116

**Published:** 2016-12-13

**Authors:** Marie Bruyneel

**Affiliations:** Chest Service, Saint-Pierre University Hospital, B-1000 Brussels, Belgium; Marie_Bruyneel@stpierre-bru.be; Tel.: +32-2-535-4219; Fax: +32-2-535-4174

**Keywords:** telediagnostics, teleconsultation, teletherapy, telemonitoring, obstructive sleep apnea, positive airway pressure

## Abstract

The use of assistive technology and telemedicine is likely to continue to shape our medical practice in the future, notably in the field of sleep medicine, especially within developed countries. Currently, the number of people suffering from obstructive sleep apnea syndrome (OSAS) is increasing. Telemedicine (TM) can be used in a variety of ways in sleep medicine: telediagnostics, teleconsultation, teletherapy and telemonitoring of patients being treated with positive pressure devices. In this review, we aim to summarize the recent scientific progresses of these techniques and their potential clinical applications and give consideration to the remaining problems related to TM application.

## 1. Introduction

During the last decade, the use of assistive technology and telemedicine (TM) has dramatically increased in developed countries. In 2005, 1090 PubMed publications were related to TM. However, by 2015 the number of papers published on this subject had increased to 2307.

TM involves bi-directional interaction between patients and healthcare providers [1]. The type of data transmitted by the patient, the frequency of data transfer, and the frequency of interactions between patient and healthcare providers varies largely across studies, according to the purpose of TM interventions [1]. Patient data transmission methods include telephone, Internet, video and smartphone data transfer. Feedback to the patients is given using the same methods.

In sleep medicine, telehealth is applicable at each stage of patient management, from diagnosis to the monitoring of treatment. In this review, we will focus on the potential use of TM in obstructive sleep apnea syndrome (OSAS). We aim to summarize the recent scientific progresses of these techniques and their potential clinical applications, and the remaining problems and barriers related to TM application. We will detail recent advances in telediagnostics, teleconsultation, teletherapy and telemonitoring in OSAS patients [2].

## 2. Telediagnostics for Obstructive Sleep Apnea Syndrome

Today, polysomnography (PSG) remains the reference method for the diagnosis of sleep disordered breathing (SDB) [3]. Patients spend one night in a sleep laboratory to enable an attended, in-lab PSG to be performed. As the number of obese people continues to rise, OSAS is becoming increasingly prevalent [4] and, as a result, the waiting time to obtain a PSG can be very long. The high cost of attended in-lab PSG (equipment, maintenance costs, staff costs and hospitalization) can be a limiting factor.

To address these problems, development of simplified portable monitoring devices (PM), to perform home sleep testing, began in the 1980s and continues today. These machines generally record up to a maximum of four cardiorespiratory signals (polygraphs), and their use has led to earlier diagnosis and treatment initiation, better patient comfort and potential cost-savings [5]. However, the lack of a skilled attendant and the patient’s inability to use a PM correctly can affect the quality of the recording: failed exams are about 10–20% [6,7]. Home-PSG, a complete sleep recording device, which includes electroencephalographic channels, avoids many of these problems, but the failure rate due to the lack of a skilled attendant continues to be a problem. With home-PSG, poor quality recordings vary between 4.7% and 20% compared to 0%–8% in the sleep lab environment [8]. TM has been studied in these circumstances in order to obtain a better quality of sleep recordings.

**Figure 1:**
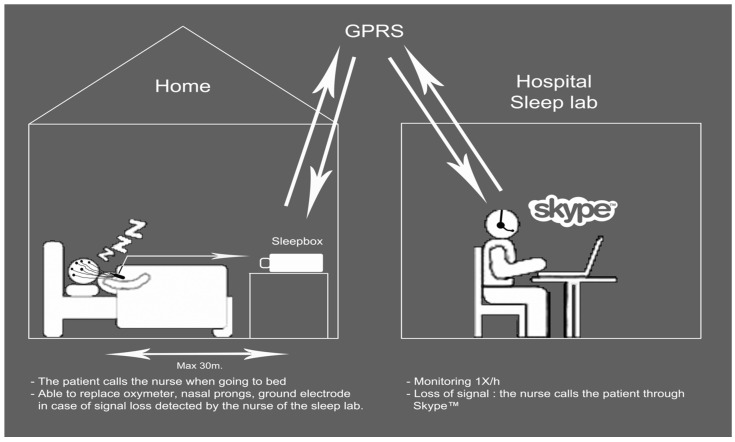
This scheme shows the working telemonitoring protocol for home-polysomnographic recording. From Bruyneel et al. [11] *Int. J. Med. Inform.* **2013**, *82*, 696–701, Copyright © 2013 with permission from Elsevier.

Three studies have tested the impact of real-time telematic transmission for unattended PSG in patients with a clinical suspicion of OSAS. Gagnadoux et al. [9] included 99 patients in a prospective randomized crossover trial. Each patient underwent one home-PSG and one in-hospital unattended, but telemonitored, PSG (TM-PSG) on two consecutive nights. The TM-PSG was recorded in the medical unit of two peripheral hospitals, with remote control from the central sleep lab. The sleep technicians checked the quality of the recordings every 30 minutes and instructed the nursing staff at the two hospitals to replace any electrodes which gave faulty signals. Failure rate was 11% for TM-PSG vs. 23% for home-PSG. Thirteen TM-PSGs required technical intervention to replace lost sensors, but in four cases, the nurse in the medical unit was not able to correct the problem. Without telemonitoring, the failure rate would have been 19%, but was reduced to 11% with remote supervision. A cost analysis was also performed and concluded that although telemedicine was more effective (half the number of failures) it was also very expensive ($244 vs. $153 for home-PSG) [10]. We have conducted two studies to assess the performance of the Sleepbox^®^ device to obtain real-time remote supervision of home or unattended PSG from the sleep lab [11,12]. The first pilot study was performed in Brussels, on 21 patients with a clinical suspicion of OSAS. Sleepbox^®^ is a wireless system able to communicate with the polysomnograph to transmit recordings to the sleep lab in real time. It also includes a microphone. The sleep technicians performed a remote monitoring of the home PSG every hour. In case of sensor loss, the technician was able to call the patient, through Skype^®^ or via the Sleepbox^®^ microphone, to ask the patient to replace the sensors correctly (Figure 1).

Ninety percent of the recordings were of excellent quality. Among the 10% PSG failure rate, one failure was due to the polysomnograph (battery failure), and one was related to a recording of poor quality. For sensor losses, two Skype^®^ interventions were required, resulting in readjustment of the defective probes. On the basis of these first encouraging results, and after some technical improvements, we performed a second study in Paris [12], to monitor real-time unattended PSG performed on 27 patients hospitalized in the acute coronary care suffering from acute coronary syndrome. The purpose was to screen OSAS in this particular population, likely to suffer frequently from SDB. The PSG was remotely controlled from the sleep lab of the same hospital, located in another building. The sleep lab nurses called their colleagues in the acute coronary care to replace probes in case of faulty signals. Results were much more interesting: all sleep recordings were interpretable and 89% of PSGs were graded as excellent. Eighty-two percent of the patients exhibited SDB. The Sleepbox^®^ was efficient in 78% of the patients and all the problems related to remote surveillance were linked to the 3G network connection. However, 10 interventions were performed: eight for replacement of the nasal cannula; one for electrode repositioning; and one for pulse oximeter, effectively increasing the global quality of PSG recordings. We did not assess the cost of the Sleepbox^®^ system.

A recent study from Coma-del-Corral et al. [13] investigated telemonitored polygraphy (TM-PG) in patients with clinical suspicion of OSAS. TM-PG was performed on 40 patients, in a “Virtual Sleep Unit”, in another hospital some 80 km from the central sleep lab. The sleep lab nurses performed real-time continuous TM-PG check. Continuous videomonitoring (via a Webcam) was also available. No PG failure was observed, but data transmission failed for 2.5% of the recordings. The cost analysis showed also that telemedicine is associated with additional costs: TM-PG cost €277 compared to €145 for a PSG.

These studies, which were conducted in different settings and on patients with various clinical conditions, have shown that real-time attended intermittent or continuous remote supervision of home/unattended PSG/PG is feasible and has the potential to reduce failure rates of sleep recordings.

## 3. Teleconsultation for Obstructive Sleep Apnea Syndrome

Teleconsultation is a system to facilitate healthcare accessibility for OSAS patients. In a recent interesting study, Isetta et al. [14] tested the feasibility of teleconsultation. Two different schemes were studied to assess whether teleconsultation could replace: (1) continuous positive airway pressure (CPAP) follow-up consultation (50 patients); (2) CPAP training consultation (40 patients randomized to receive face-to-face vs. teleconsultation). For CPAP follow-up, 95% of the patients were satisfied with the teleconsultation, and 66% declared that teleconsultation could replace 50–100% of the CPAP therapy follow-up visits. Younger patients (<65 years) were more inclined to recommend teleconsultation to others. For CPAP training, patients trained via videoconference demonstrated the same knowledge about OSAS and CPAP therapy as the face-to-face group (94% of correct answers vs. 92%). Video-trained patients also showed similar performances on mask placement and mask leak avoidance.

Coma-del-Corral et al. [13] implemented teleconsultation in patients with confirmed OSAS. After the TM-PG, patients were randomized to receive either a face-to-face consultation or a teleconsultation to receive the results of their sleep study. The teleconsultation was made through videoconferencing. The 16 patients requiring CPAP were then treated at home by auto positive airway pressure device (APAP) and the data was telematically transmitted during two nights. At six months, in this very small group of patients, adherence was not different: 85% for the face-to-face consultation and 75% for the teleconsultation group.

In these two prospective studies, teleconsultation seems to be an interesting and viable option for the purpose of CPAP education and follow-up.

## 4. Teletherapy with CPAP for Obstructive Sleep Apnea Syndrome

In order to perform remote-attended CPAP titration at home, Dellaca et al. [15] recorded 20 severe OSAS patients who were using CPAP for the first time. CPAP was coupled with a telemetric unit, working via General Packet Radio Services (GPRS) mobile phone network in order to allow remote control of CPAP parameters (flow, pressure, leaks) and CPAP pressure adaptation. One week later, patients underwent full in-lab PSG and another CPAP titration. Pressure level was similar in both settings: 9.15 at home vs. 9.2 cm H_2_O. 

Real-time remote CPAP titration is feasible and offers pressure-setting outcomes similar to in-hospital attended CPAP titration.

## 5. Telemonitoring of CPAP-Treated Obstructive Sleep Apnea Syndrome Patients

When treating OSAS patients with CPAP, the challenge is to obtain adequate adherence, defined as use during at least 4 h/night and for more than 70% of the nights [16]. Independently of this usually accepted cut-off, the CPAP effect grows with increased use. Weaver et al. [17] showed a linear relationship between CPAP use and subjective/objective sleepiness. Using the Functional Outcomes of a Sleep Questionnaire, these authors showed a greater improvement in memory when CPAP was used more than 6 h/night in comparison with <2 h. Barbé et al. [18] demonstrated, in a series of 359 OSAS patients, that nightly use longer than 5.65 h achieved better blood pressure and sleepiness reduction. A recent randomized study from Bouloukaki et al. [19] in a cohort of 3100 CPAP-treated patients, randomized in intensive versus standard interventions, also confirmed the positive effect of a greater CPAP use (6.9 vs. 5.2 h/night) on cardiovascular outcomes, indicating that a regular 5–6 h use/night is required. It has also been demonstrated that early adherence (at one week or one month) is associated with better adherence at six months [20,21]. In a recent literature review, the long-term adherence rate has been estimated at 66% [22]. Factors such as psychological barriers, social concerns, side effects, disease characteristics and first CPAP exposure can affect treatment acceptance and adherence [23] and as many as 5 to 50% of patients refuse treatment or reject it rapidly after initiation [24]. Improvements can be obtained through supportive, educational and behavioral therapy. A recent Cochrane Database Systematic Review [25] pooling data from 30 low-to-moderate quality studies reported that supportive, educational and behavioral therapy increases adherence by a respective amount of 50, 35 and 104 min/night and also results in a larger proportion of patients using CPAP for more than 4 h/night.

During the last decade, TM has been applied in order to improve adherence. In all the studies, CPAP devices were fitted with a wireless data transmitter to collect compliance and efficacy data. The way to deliver feedback/interventions to the patients in case of troubleshooting varies between studies. The randomized studies comparing TM follow-up versus standard care for CPAP patients are shown in Table 1.

We can see that, despite the use of globally similar methods and a reasonably large number of patients, the results are disappointing. Half of the studies do not show any improvement in adherence with telemonitoring. We must emphasize that in two of these studies [28,29], despite the telemonitoring, the adherence remained very low, questioning the value of the usual care in these series.

A recent study has assessed the impact of direct access to daily CPAP device parameters for patients. Kuna et al. [34] randomized 138 recently diagnosed OSAS patients requiring CPAP, to usual care, usual care with access to CPAP usage, or usual care with access to CPAP usage and a financial incentive. After three months, mean adherence was 4.8 and 5 h/night in the intervention groups vs. 3.8 for usual care (*p* < 0.0001). Web access and direct daily feedback seems to act positively on adherence. Interestingly, patients frequently consulted their own data during the first week of treatment, but this then decreases rapidly. More CPAP data consultation was associated with better CPAP adherence.

Telemonitoring for CPAP-treated OSAS patients is currently widely applied. Even if the impact on adherence is limited, it offers the possibility for the healthcare providers to detect “problematic” patients and to react accordingly. However, multimedia approaches offer other advantages as it can help save nursing time [31,32], which could allow more patients to be managed with the same manpower.

## 6. Discussion

Sleep medicine has always relied heavily on technology, and telemedicine now offers more possibilities. In 2013 already, a systematic review focused on teleneurology stressed, through the very pragmatic Functionality, Application, Technology, Evaluative phase (F.A.T.E.) scoring system, the emergence of little but solid literature regarding sleep disorders [35]. The present review has demonstrated that numerous telemedicine options are available to enhance ambulatory care, healthcare accessibility and remote therapy monitoring for OSAS patients. However, some points of care are less developed than others.

Telediagnostics for OSAS is not yet widely implemented. This can be explained by the fact that even if telediagnostics is efficacious in offering better quality, more comfort and enhanced accessibility to sleep tests, its widespread use is slowed down by the costs and the complexity of the technical aspects. It also requires a change to the current model of care delivery, as it will become home- and patient-centered rather than hospital-centered. Such huge changes are going to take time to be implemented.

Teleconsultation seems to be easier to practice, since it does not require changes in the model of care, just a good teleconference platform. This method is associated with numerous advantages for patients including the removal of the need to travel to and from the healthcare center. Teleconsultation is a part of sleep-integrated models of care and some centers, in the United States, have longstanding experience in teleconsultation-guided care [36,37]. Baig et al. [36] demonstrated the long-term effectiveness of a five-year TM program: the delay to obtain CPAP was reduced from more than two months to less than one week. 

The concept of remote-attended CPAP titration at home is very attractive, but questions remain. As home-APAP titration is nowadays currently performed with good results [38,39,40,41], one can wonder if it is really necessary to obtain a remote real-time control of titration. With unattended home-APAP titration, the treatment can quickly be adjusted following analysis of the downloaded data from the CPAP device. Currently, patients have to visit the sleep lab shortly after home-APAP titration and, therefore, a remote-attended titration strategy would simplify this. Costs of both strategies should be assessed in future studies. The problem of performing home-APAP titration is more related to the contra-indications than to technical aspects. This pathway is restricted to patients without comorbidities (neuropsychiatric, cardiovascular, respiratory comorbidities and comorbid sleep disorders) [42] and with a body mass index (BMI) below 40. In our local experience, the proportion of patients who can benefit from home-APAP titration is about 30%. We also know from previous studies that even in well-selected patients, APAP titration failure occurs in 6–15% of cases [38,39,40,41]. Home-APAP titration is very interesting for selected patients, who will benefit from treatment in more familiar surroundings.

Telemonitoring for CPAP-treated OSAS patients is clearly the most popular of TM tools in sleep medicine. The transmission system for CPAP data is universally available in recent models of machines. Telemonitoring allows better control of therapy. The sleep lab team will quickly be able to distinguish problematic patients requiring more support, education and time investment. Telemonitoring will also avoid unnecessary nurse/medical visits to correctly treated patients. The impact on adherence is uncertain but recent data highlighting nurse saving time shows another added value of TM monitoring [31,32].

The benefits of TM in OSAS patients have been demonstrated through several studies, for different stages of care. Telemonitoring is widely implemented and helps both clinicians and patients to, not only monitor, but to accurately and rapidly adjust the CPAP therapy. Teleconsultation use is also likely to grow in the future. These two aspects of OSAS care do not require large changes in care programs or strategies. Contrarily, remote continuous attendance of sleep tests and home-APAP titration require more technological and human resources together with a change in patient care management. In my opinion, according to the turning of hospital strategies to offer more ambulatory care [43], TM is going to evolve and it is likely that there will be an increasing development of tools and activities in all areas of sleep medicine, including remote continuous attendance of sleep tests and home-APAP. 

Despite these positive aspects, there are some limitations related to TM. Research findings were not able to show an improvement of CPAP adherence with telemonitoring, but there were marked savings in nurse time in two of the studies [31,32]. In future studies, other outcomes of telemonitoring should be assessed, such as cost savings, cost-effectiveness, long-term clinical control of comorbidities, etc. Secondly, TM is expensive, and this is related to the complex technology required to implement telehealth. Costs could be decreased by wider use of TM in the future. There are also still privacy concerns related to TM use. Privacy protection and security of medical data transmission are two key points to be strictly regulated and controlled to avoid ethical problems. Work is in progress, as regulations for TM deployment for European healthcare have been published last year [44]. In the United States, efforts have also been made, in many states, to regulate, but also to bill, telehealth [45,46]. To end, few research teams are reporting technical problems related with the use of TM. Medicine and automatization are two different worlds that do not usually coincide. How do you perform a teleconsultation if you are unable to log in on the platform or you experience a bug with your webcam, if CPAP data is not available on the secured platform? We know that informatics are excellent tools when working correctly, but real-life experiences demonstrate the need for an efficient helpdesk in order for the system to work proficiently. Therefore, it is apparent that TM will never be suitable for all patients.

## 7. Conclusions

There is growing evidence to support the implementation of TM in OSAS patients. The most studied tools include telemonitoring in CPAP-treated patients and teleconsultation. The impact of telemonitoring on short-term adherence is uncertain, but nurse saving time has been demonstrated. There are still some barriers to the implementation of remote attendance for sleep tests and home-APAP titration, but it is likely to change with the current trend to offer more outpatient care. Since TM will shape the clinical landscape of tomorrow, clinicians will have to adapt their practice to face technological progress whilst taking into account the limitations of these techniques.

## Figures and Tables

**Table 1:** Results of the randomized studies comparing telemedicine follow-up versus standard care for CPAP patients. AHI: apnea-hypopnea index; TM: telemedicine; CPAP: continuous positive airway pressure; S: statistically significant; NS: not statistically significant.

	*N* =	AHI	TM Methods	Adherence TM vs. Usual Care
Taylor et al. 2006 [26]	114	>5	Internet support + feedback.	1 month: 4.3 vs. 4.2 h/N (NS)
Stepnowsky et al. 2007 [27]	45	>15	Interactive web-site with own CPAP data and guide for troubleshooting.	2 months: 4.1 vs. 3.4 h/N (S)
Sparrow et al. 2010 [28]	250	>10	Interactive voice response system (phone).	6 months: 2.4 vs. 1.48 h/N (S)
Fox et al. 2012 [29]	75	>15, mean: 42	Feedback by phone.	3 months: 3.2 vs. 1.7 h/N (S)
Isetta et al. 2015 [30]	139	Mean: 49	Feedback by web tools.	6 months: 4.4 vs. 4.2 h/N (NS)
Anttalainen et al. 2016 [31] partially randomized	111	Mean: 34	Nurse adjustment phone/visits.	12 months: 6.4 vs. 6.1 h/N (NS)
Munafo et al. 2016 [32]	132	Mean: 34 (TM group), 27 (usual care)	Multimedia approach to contact patient about their CPAP use.	1 month: 5.1 vs. 4.7 h/N (NS)
Frasnelli et al. 2016 [33] Patients selected at random	223	Median: 37 (TM group), 40 (usual care)	Feedback by phone.	1 month: 5.3 vs. 4.6 h/N (S)
